# Structural Brain Changes in Patients with Congenital Anosmia: MRI-Based Analysis of Gray- and White-Matter Volumes

**DOI:** 10.3390/diagnostics15151927

**Published:** 2025-07-31

**Authors:** Shun-Hung Lin, Hsian-Min Chen, Rong-San Jiang

**Affiliations:** 1 Department of Otolaryngology, Taichung Veterans General Hospital, Taichung 407219, Taiwan; inty080@vghtc.gov.tw; 2 Center for Quantitative Imaging in Medicine (CQUIM), Department of Medical Research, Taichung Veterans General Hospital, Taichung 407219, Taiwan; hsmin@vghtc.gov.tw; 3 Department of Otolaryngology, Tungs’ Taichung MetroHarbor Hospital, Taichung 435403, Taiwan; 4 Department of Medical Research, Taichung Veterans General Hospital, Taichung 407219, Taiwan; 5 School of Medicine, Chung Shan Medical University, Taichung 40201, Taiwan

**Keywords:** congenital anosmia, gray matter, hemisphere asymmetry, magnetic resonance imaging, white matter

## Abstract

**Background:** Congenital anosmia (CA) is a rare condition characterized by a lifelong inability to perceive odors, which significantly affects daily life and may be linked to broader neurodevelopmental alterations. This study aimed to investigate structural brain differences in patients with CA using MRI, focusing on gray matter (GM) and white matter (WM) changes and their implications for neurodevelopment. **Methods:** This retrospective study included 28 patients with CA and 28 age- and gender-matched healthy controls. Patients with CA were diagnosed at a single medical center between 1 January 2001 and 30 August 2024. Controls were randomly selected from an imaging database and had no history of olfactory dysfunction. Brain Magnetic Resonance Imaging (MRI)was analyzed using volumetric analysis in SPM12.GM and WM volumes were quantified across 11 anatomical brain regions based on theWFU_PickAtlas toolbox, including frontal, temporal, parietal, occipital, limbic, sub-lobar, cerebellum (anterior/posterior), midbrain, the pons, and the frontal–temporal junction. Left–right hemispheric comparisons were also conducted. **Results:** Patients with CA exhibited significantly smaller GM volumes compared to healthy controls (560.6 ± 114.7 cc vs. 693.7 ± 96.3 cc, *p* < 0.001) but larger WM volumes (554.2 ± 75.4 cc vs. 491.1 ± 79.7 cc, *p* = 0.015). Regionally, GM reductions were observed in the frontal (131.9 ± 33.7 cc vs. 173.7 ± 27.0 cc, *p* < 0.001), temporal (81.1 ± 18.4 cc vs. 96.5 ± 14.1 cc, *p* = 0.001), parietal (52.4 ± 15.2 cc vs. 77.2 ± 12.4 cc, *p* < 0.001), sub-lobar (57.8 ± 9.7 cc vs. 68.2 ± 10.2 cc, *p* = 0.001), occipital (39.1 ± 13.0 cc vs. 57.8 ± 8.9 cc, *p* < 0.001), and midbrain (2.0 ± 0.5 cc vs. 2.3 ± 0.4 cc, *p* = 0.006) regions. Meanwhile, WM increases were notable in the frontal(152.0 ± 19.9 cc vs. 139.2 ± 24.0 cc, *p* = 0.027), temporal (71.5 ± 11.5 cc vs. 60.8 ± 9.5 cc, *p* = 0.001), parietal (75.8 ± 12.4 cc vs. 61.9 ± 11.5 cc, *p* < 0.001), and occipital (58.7 ± 10.3 cc vs. 41.9 ± 7.9 cc, *p* < 0.001) lobes. A separate analysis of the left and right hemispheres revealed similar patterns of reduced GM and increased WM volumes in patients with CA across both sides. An exception was noted in the right cerebellum-posterior, where patients with CA showed significantly greater WM volume (5.625 ± 1.667 cc vs. 4.666 ± 1.583 cc, *p* = 0.026). **Conclusions:** This study demonstrates widespread structural brain differences in individuals with CA, including reduced GM and increased WM volumes across multiple cortical and sub-lobar regions. These findings suggest that congenital olfactory deprivation may impact brain maturation beyond primary olfactory pathways, potentially reflecting altered synaptic pruning and increased myelination during early neurodevelopment. The involvement of the cerebellum further implies potential adaptations beyond motor functions. These structural differences may serve as potential neuroimaging markers for monitoring CA-associated cognitive or emotional comorbidities.

## 1. Introduction

Congenital anosmia (CA) is a rare but significant olfactory dysfunction characterized by a lifelong absence of odor perception [1,2,3], with an estimated prevalence of approximately 1 in 10,000 individuals [4]. This sensory loss not only impairs quality of life but has also been linked to structural alterations in regions such as the prefrontal cortex and limbic system [5], as well as to compensatory functional activation in olfactory-related cortical regions, with patients with CA showing increased posterior orbitofrontal cortex and insula responses during the expectancy of and reading of odor-associated words [6]. With the advancement of neuroimaging techniques, Magnetic Resonance Imaging (MRI) has become a crucial tool for investigating structural and functional changes in the brains of patients with CA [7,8]; for example, previous studies have performed volumetric analyses using high-resolution T1-weighted MRI scans [9]. Individuals with CA commonly exhibit structural abnormalities in the olfactory pathways and associated brain regions, including absence or hypoplasia of the olfactory bulb [5] and significantly reduced gray matter volume in the bilateral olfactory sulci [10], offering important implications for understanding the neurodevelopmental basis of CA [5,11,12,13,14,15,16]. These findings provide essential insights into the pathological mechanisms of CA. However, the research on brain structural changes in CA remains limited, especially regarding the application of diverse MRI techniques in this context. Most existing studies have focused on isolated olfactory regions or employed small sample sizes—ranging from 16 to 36 participants [9,17,18,19]—which constrains statistical power and limits the generalizability of their findings.

Prior imaging studies of congenital sensory deprivation have demonstrated increased cortical thickness in the primary visual cortex in early blindness [20] and in the left piriform and entorhinal cortices in congenital deafness [19], implying a generalized neural response to early sensory deprivation [19,21]. In contrast, most studies on acquired anosmia suggest a different pattern, often involving localized gray matter atrophy rather than global structural reorganization [22,23,24]. This distinction emphasizes the importance of studying congenital cases separately as their effects on neurodevelopment may be qualitatively different from those arising later in life [25].

To address these limitations, in this study we aimed to employ a whole-brain volumetric analysis approach to comprehensively assess structural brain alterations in CA. This approach allows for the detection of subtle and widespread structural differences beyond traditional olfactory regions, which may reflect long-term developmental consequences of lifelong olfactory deprivation on brain organization. By investigating both gray- and white-matter changes, and incorporating hemispheric comparisons, this study seeks to reveal how congenital olfactory loss may influence the architecture of the brain beyond primary sensory areas. We hypothesize that structural alterations in congenital anosmia are not confined to the traditional olfactory regions but also involve hemispheric asymmetries and broader brain systems. Furthermore, exploratory whole-brain analyses may reveal additional morphological changes in regions involved in sensory integration and cognitive control, reflecting potential cross-modal plasticity and broader adaptations to lifelong olfactory deprivation.

## 2. Materials and Methods

### 2.1. Inclusion Criteria

Participants were included in this study based on the following strict criteria: (1) a detailed and structured medical history indicating the absence of olfactory perception since childhood; (2) anosmia confirmed by assessing birhinal odor-detection thresholds using the phenyl ethyl alcohol (PEA) threshold test—participants with a birhinal threshold of –1 were classified as anosmic [26]; and (3) MRI findings demonstrating either non-visualization or hypoplasia of the olfactory bulb. Healthy controls with no history of olfactory dysfunction were randomly selected from our image database and matched to participants by age (±2 years) and gender. All MRI scans of control participants were interpreted as showing a normal contour of the olfactory bulb.

### 2.2. Exclusion Criteria

The following exclusion criteria were applied to both patients and controls: (1) clinical signs or symptoms indicative of secondary anosmia, including severe chronic rhinosinusitis, traumatic brain injury, or other known causes of olfactory impairment; (2) structural abnormalities in the anterior skull base or paranasal sinuses resulting from surgical intervention or other pathologies (e.g., tumors); and (3) brain MRI acquired before the age of 20 since our IRB approval covered only adult recruitment and our institutional normative fluid-attenuated inversion recovery(FLAIR) dataset includes participants aged 20 years and older.

### 2.3. Participants

We retrospectively collected patients with CA diagnosed at a single medical center between 1 January 2001 and 30 August 2024. Fifty-eight cases met the inclusion criteria. Thirty patients were excluded based on exclusion criteria or incomplete data. Additionally, 28 healthy individuals with no history of anosmia, age- and gender-matched, were randomly selected from our image database retrospectively to serve as the control group. A total of 56 participants were included in the final analysis: 28 individuals with CA (16 women, 12 men; age range: 20–60 years; mean age: 32.71 years, SD: 14.02) and 28 healthy controls (14 women, 14 men; mean age: 32.93 years, SD: 14.03).

All participants underwent axial FLAIR imaging, acquired with the following parameters: repetition time (TR)/inversion time (TI) = 9000–10,000 ms/2400–2700 ms, echo time (TE) = 86–121 ms, 1 excitation, with variable flip-angle distribution, slice thickness = 6 mm, matrix 290 × 224, and field of view= 22–24 cm. Imaging was performed using a Siemens 1.5T scanner (Aera, Siemens, Erlangen, Germany) for all control participants and twenty-three patients; five patients were scanned using a Philips 3T scanner (Ingenia CX, Philips Healthcare, Best, The Netherlands). Despite scanner heterogeneity, all images were acquired using FLAIR sequences covering the entire brain and were processed using a consistent preprocessing and tissue segmentation pipeline (see Section 2.4). Visual quality control was performed by two authors (S.-H.L. and H.-M.C.) to ensure stable segmentation performance across platforms.

For each participant, gray matter (GM) and white matter (WM) volumes were quantified across 11 predefined brain regions using SPM12 software version 7487. These regions included frontal, temporal, limbic, parietal, sub-lobar, occipital, midbrain, frontal–temporal, cerebellum–anterior, cerebellum–posterior, and the pons. This study was approved by the Institutional Review Board (III) of Taichung Veterans General Hospital (Approval No. CE24527C) and involved medical records from the Smell and Taste Center of the Department of Otorhinolaryngology.

### 2.4. SPM12

MRI images of all participants were analyzed. FLAIR images were selected for gray- and white-matter segmentation because our institutional anosmia MRI protocol includes FLAIR sequences covering the entire brain, whereas T1-weighted acquisitions are limited to frontal base slices. Previously, FLAIR-only automated segmentation was demonstrated to produce volumetric measurements consistent with T1-based methods [27]. Importantly, for each of the 28 control subjects, segmentation outputs were visually inspected slice-by-slice against the native FLAIR images to confirm anatomical plausibility. A retrospective review excluded any scans with excessive noise, motion artifacts, or other quality issues; no congenital anosmia case images required exclusion following this quality-control process.

**Figure 1:**
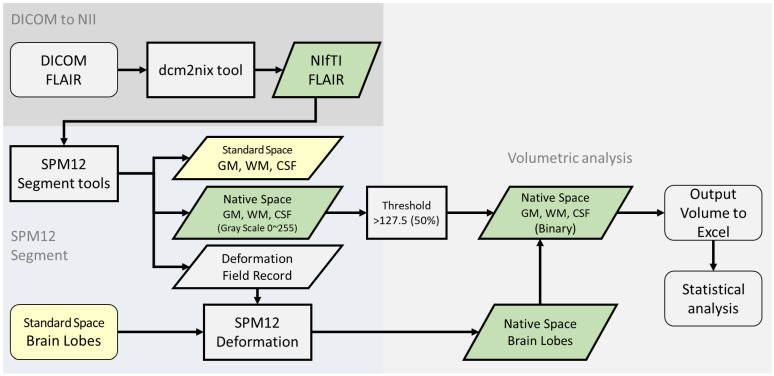
Illustration of the workflow for brain tissue segmentation from MR images using the methods available in SPM12.

The FLAIR images were first converted from DICOM format to NIfTI format (Neuroimaging Informatics Technology Initiative) using the dcm2niix tool [28]. We then used the open-source and free Statistical Parametric Mapping (SPM12) software (updated in 2020), developed by The Wellcome Centre for Human Neuroimaging at UCL, for image segmentation [29,30] (Figure 1).

**Figure 2:**
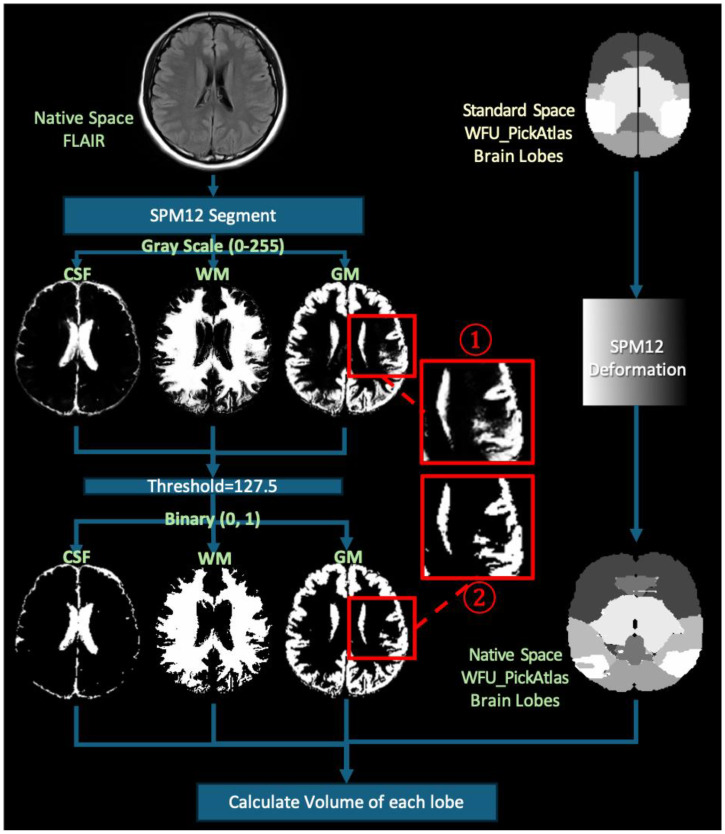
Illustration of the workflow for brain tissue segmentation from MR images using the methods available in SPM12.The red-outlined region (1) shows an enlarged view of the grayscale segmentation result, whereas in the lower panel, the red-outlined region (2) displays the corresponding binarized (thresholded) segmentation mask.

Using the “Segment” function in SPM12, we segmented all MRI images into GM, WM, and cerebrospinal fluid. For the segmentation parameters, we selected the ICBM space template—East Asian brains—and output images in the original space to avoid interpolation errors. The segmentation pipeline implemented in SPM12 inherently includes several robust preprocessing steps, such as bias field correction, tissue probability mapping, and spatial normalization using an iterative expectation–maximization algorithm. These integrated procedures help to reduce scanner-related intensity inhomogeneity and anatomical variability, thereby enhancing the stability and consistency of gray- and white-matter classification across different imaging platforms. After segmentation, we obtained GM, WM, and cerebrospinal fluid probability maps in the original space by applying a tissue-probability threshold of 0.5, following the convention of the original unified segmentation framework in SPM12 [30], consistent with other volumetric studies [31,32]. These binary maps were used for tissue quantification. Visual quality control was conducted on all segmentation outputs to ensure anatomical plausibility and consistency (Figure 1 and Figure 2).

Additionally, during the segmentation process, the deformation fields generated by SPM12 software version 7487 were retained. Using the Deformation Field tool in SPM12, standard-space brain templates—specifically, the Talairach Daemon (TD) Brain Lobes—were transformed into the subject’s original image space. The TD Brain Lobes are part of the WFU_PickAtlas toolbox (RRID:SCR_007378), which is an automated anatomical labeling resource based on the Talairach coordinate system. Using SPM12, the TD Brain Lobes template was applied to divide the brain into lobar regions, such as the frontal, parietal, temporal, and occipital lobes.

In our study, we used the WFU_PickAtlas templates to segment the brain into either 11 major regions (without distinguishing left and right hemispheres) or 22 regions (with left/right separation).

### 2.5. Power Analysis and Sample Size Estimation

Power analysis was performed by G*Power Software version 3.1.9.7. A total sample size of 56 participants would provide 83% power for analysis of variance with two groups to detect significant differences with a 0.81 effect size at the α = 0.05 significance level.

### 2.6. Statistical Analyses

All statistical analyses were performed using SPSS (version 22; IBM Corp., Armonk, NY, USA). Continuous variables are presented as means ± standard deviations. Given the non-normal distribution of volumetric data, group comparisons between patients with CA and healthy controls were conducted using the non-parametric Mann–Whitney U test. A *p*-value less than 0.05 was considered statistically significant. Group comparisons were performed for total and regional GM and WM volumes, including bilateral comparisons across 11 predefined brain regions. Additionally, GM and WM proportions relative to total brain volume were calculated and compared between groups.

## 3. Results

### 3.1. Overall Brain Volumes

Patients with CA exhibited significantly smaller GM volumes compared to healthy controls (*p* < 0.001) but larger WM volumes (*p* = 0.015). When analyzed as percentages of total brain volume, the proportion of GM was also significantly reduced in patients with CA (*p* < 0.001), whereas the proportion of WM was higher (*p* < 0.001) (Table 1).

### 3.2. Gray Matter

Table 2 shows a comparison of GM volume in 11 brain regions between patients with CA and healthy controls. Significant GM reductions were found in the frontal (*p* < 0.001), temporal (*p* = 0.001), parietal (*p* < 0.001), sub-lobar (*p* = 0.001), occipital (*p* < 0.001), and midbrain (*p* = 0.006) regions in patients with CA.

To explore lateralized group differences, we conducted a comparison of GM volumes in the left and right hemispheres. Bilateral GM reductions were observed in multiple brain regions. These included the frontal lobes (right: *p* < 0.001; left: *p* < 0.001), temporal lobes (right: *p* = 0.004; left: *p* = 0.001), parietal lobes (right: *p* < 0.001; left: *p* < 0.001), occipital lobes (right: *p* < 0.001; left: *p* < 0.001), midbrain (right: *p* < 0.001; left: *p* = 0.045), and sub-lobar regions (right: *p* < 0.001; left: *p* = 0.004).However, the reduction in the left midbrain did not remain significant after adjustment for multiple comparisons (adjusted *p* = 0.083) (Table 3).

### 3.3. White Matter

WM analysis revealed significantly larger volumes in patients with CA compared to healthy controls in the frontal(*p* = 0.027), temporal (*p* = 0.001), parietal (*p* < 0.001), and occipital (*p* < 0.001) regions. Notably, the difference in the frontal lobes did not remain significant after adjustment for multiple comparisons (adjusted *p* = 0.074) (Table 4).

To examine hemispheric differences, we compared WM volumes between patients with CA and healthy controls. Patients with CA showed significantly greater WM volume in the temporal lobes (right, *p* = 0.002; left, *p* = 0.001), parietal lobes (bilateral, *p* < 0.001), occipital lobes (bilateral, *p* < 0.001), and left cerebellum–posterior (*p* = 0.026). After FDR correction, the increase in the frontal lobes did not remain significant (right, adjusted *p* = 0.072; left, adjusted *p* = 0.081), nor did the increase in the left cerebellum–posterior (adjusted *p* = 0.072) (Table 5).

## 4. Discussion

This study employs a whole-brain volumetric MRI approach to investigate structural changes in congenital anosmia, extending analysis beyond traditional olfactory regions—such as the olfactory bulb, sulcus, and related cortices—to assess gray- and white-matter alterations across the entire brain. We found significant differences in gray- and white-matter volumes in patients with CA, specifically a reduction in GM volume and an increase in WM volume by SPM12. Patients with congenital anosmia showed widespread gray matter reductions—in the frontal, temporal, parietal, occipital, sub-lobar, and right midbrain regions—that overlap with the frontoparietal control network. The frontoparietal control network regulates attention, executive function, and emotional balance, and its disruption has been linked to diverse mental illnesses [33]. Lifelong olfactory deprivation may therefore weaken this “cognitive control hub,” highlighting it as a promising focus for future studies of brain health and resilience. Notably, the temporal and parietal cortices—key hubs for multisensory integration [34,35]—exhibited both gray matter reduction and white matter expansion. Future work should investigate how reduced olfactory input influences the development and functional connectivity of these multisensory regions. These findings highlight the widespread impact of congenital olfactory deprivation on brain structure.

Importantly, our findings extend beyond the traditionally defined olfactory regions, suggesting that congenital sensory deprivation might exert widespread effects on global brain architecture. The observed structural alterations across multiple cerebral lobes and the cerebellum imply that the brain’s response to the absence of sensory input is not confined to primary or secondary olfactory areas but instead reflects a broader reorganization of neural networks. This supports the notion that CA—like other forms of early sensory deprivation—may reflect compensatory or developmental changes in regions not directly associated with the lost modality, demonstrating the brain’s remarkable plasticity during early developmental stages.

Previous studies have shown that patients with CA exhibited reduced GM volume in the left medial orbitofrontal cortex [5] and bilateral olfactory sulci [10]. The latter findings were attributed to a decreased cortical area, curvature, and sulcus depth. Regarding secondary olfactory areas associated with other etiologies of anosmia, studies have consistently identified GM atrophy in regions such as the right insular cortex, left anterior cingulate cortex, and orbital frontal cortex [22,23,36,37]. For instance, Peng, P. et al. [23] observed atrophy in the right insular cortex, left anterior cingulate cortex, and left orbital frontal cortex, while Bitter, T. et al. [22,37] reported similar findings in the right insular cortex and right orbital frontal cortex. Additionally, Bitter et al. and Yao et al. [22,36,37] identified a consistent pattern of GM volume reduction in the right orbital frontal cortex. These findings align with our results, further supporting the reduction in GM volume in these regions.

Conversely, patients with CA exhibit increased GM volumes in key regions of the olfactory system compared to healthy controls and individuals with acquired anosmia. These regions include primary olfactory structures, such as the left entorhinal cortex and piriform cortex [5,19], as well as secondary olfactory areas, including the orbitofrontal cortex and insular cortex [19]. Additionally, structural differences have been identified in other olfactory-related areas, such as the middle frontal gyrus, medial orbital gyrus, and superior longitudinal fasciculus [5,10,19]. Findings related to the lateralization of these changes differ among studies, although they highlight significant directions for future research. The discrepancies among studies may stem from methodological differences, including the use of whole-brain voxel-based analysis versus region-of-interest approaches, differentMRI sequences(e.g., T1-weighted vs. FLAIR), or variations in image processing pipelines(e.g., SPM12 vs. FreeSurfer). Although previous congenital anosmia studies have employed diverse methodologies (T1-weighted VBM vs. ROI-based analyses and T1 vs. FLAIR sequences), our reliance on FLAIR imaging may influence segmentation accuracy and comparability. Segmentation outputs were visually inspected slice-by-slice by two independent raters (S.-H.L. and H.-M.C.) to ensure consistent performance across platforms, and FLAIR-only automated segmentation has been validated against high-resolution T1-weighted methods, demonstrating comparable volumetric accuracy [27]. These differences underscore the need for standardization and careful interpretation when comparing findings across studies in the field of neuroimaging related to sensory deprivation.

Accumulating evidence indicates that patients with CA exhibit increased GM in areas including the left superior longitudinal fasciculus and the superior temporal sulcus [19]. The mechanisms underlying WM changes resulting from congenital sensory deprivation remain incompletely understood. Prior studies on congenital sensory dysfunction in the visual and auditory systems have revealed similar patterns of structural alteration [20,38,39,40]. It has been suggested that sensory loss or dysfunction during early developmental stages is associated with increased cortical thickness in certain core structures [19,21]. This phenomenon is thought to stem from mechanisms distinct from those involved in acquired sensory loss. One plausible explanation is the complete absence of sensory input during cortical development. The findings suggest that these alterations may be attributed to brain development processes, particularly the experience-dependent pruning of axons and synapses, which are more pronounced during early life [21,41]. Our findings also showed an increase in WM volume, aligning with previously reported results.

This study has several limitations. First, our retrospective, single-center cohort of 56 participants (28 patients with CA, 28 controls) is relatively modest, which may reduce the statistical power and introduce selection bias. Second, despite thorough SPM-based preprocessing and slice-by-slice visual quality control by the authors, our exclusive reliance on FLAIR sequences may still introduce residual measurement variability. Third, while this study focused on structural differences in GM and WM volumes, it did not account for subregional variations within olfactory brain areas. Subregion-specific analyses could provide deeper insights into how CA uniquely affects individual components of the olfactory system. Incorporating these analyses in future studies would enhance the precision and interpretability of findings. Second, brain structure is influenced by a multitude of factors beyond olfactory input, including cognitive abilities, educational background, environmental experiences, and handedness. These factors were not fully accounted for in this study and might have confounded the observed structural differences. For instance, handedness is known to influence brain lateralization and could affect the interpretation of results related to hemispheric differences [42]. Finally, we did not calculate associations between our volumetric measures and clinical variables (e.g., olfactory threshold scores or disease duration), limiting our ability to link structural changes to functional impairment. Further research should include comprehensive assessments of these variables to disentangle their contributions from the effects of congenital sensory deprivation.

## 5. Conclusions

This study, utilizing a whole-brain MRI approach, revealed significant structural brain alterations in patients with CA, characterized by a global reduction in GM volume and a corresponding increase in WM volume compared to healthy controls. These volumetric differences were predominantly observed in the frontal, temporal, parietal, occipital, and sub-lobar regions, suggesting a widespread impact of congenital olfactory deprivation on brain development beyond traditionally defined olfactory areas. The increased WM volumes, particularly in regions corresponding to GM reductions, might reflect compensatory mechanisms or altered developmental trajectories. Overall, the bilateral and regional alterations in GM and WM observed in this study point toward a more generalized and complex neurodevelopmental impact of congenital anosmia than has been previously recognized. Future studies should employ subregional and clinical analyses, consider cognitive and experiential factors, and use multimodal neuroimaging to clarify CA’s structural and functional impacts.

## Figures and Tables

**Table 1:** Comparison of whole brain volumes between patients with CA and healthy controls.

	Patients with CA	Controls	*p*-Value
Volume			
Whole brain	1104.9 ± 114.4 (cc)	1184.8 ± 158.4 (cc)	0.071
Gray matter	560.6 ± 114.7 (cc)	693.7 ± 96.3 (cc)	<0.001
White matter	554.2 ± 75.4 (cc)	491.1 ± 79.7 (cc)	0.015
Percentage			
Gray matter	50.45 ± 7.22 (%)	58.60 ± 3.14 (%)	<0.001
White matter	49.55 ± 7.22 (%)	41.4 ± 3.14 (%)	<0.001

CA: congenital anosmia.

**Table 2:** Comparison of gray matter volumes between patients with CA and healthy controls.

Area	Patients with CA (cc)	Controls (cc)	Effect Size	*p*-Value	Adjusted *p*-Value †
Frontal	131.9 ± 33.7	173.7 ± 27.0	0.56	<0.001	<0.001
Temporal	81.1 ± 18.4	96.5 ± 14.1	0.43	0.001	0.002
Limbic	59.3 ± 8.9	61.9 ± 9.1	0.11	0.422	0.464
Parietal	52.4 ± 15.2	77.2 ± 12.4	0.69	<0.001	<0.001
Sub-lobar	57.8 ± 9.7	68.2 ± 10.2	0.46	0.001	0.002
Occipital	39.1 ± 13.0	57.8 ± 8.9	0.65	<0.001	<0.001
Midbrain	2.0 ± 0.5	2.3 ± 0.4	0.37	0.006	0.011
Frontal–Temporal	0.2 ± 0.2	0.2 ± 0.1	0.05	0.731	0.731
Cerebellum–Anterior	30.7 ± 4.5	32.3 ± 4.6	0.14	0.287	0.395
Cerebellum–Posterior	37.1 ± 6.1	39.0 ± 5.1	0.12	0.376	0.460
Pons	0.6 ± 0.3	0.8 ± 0.2	0.22	0.098	0.154

CA: congenital anosmia. † Benjamini–Hochberg method.

**Table 3:** Comparison of right/left gray matter volumes between patients with CA and healthy controls.

Area	Hemisphere	Patients with CA (cc)	Controls (cc)	Effect Size	*p*-Value	Adjusted *p*-Value †
Frontal	R	66.5 ± 16.7	86.8 ± 13.2	0.56	<0.001	<0.001
	L	64.8 ± 16.9	85.8 ± 13.8	0.56	<0.001	<0.001
Temporal	R	39.6 ± 8.5	46.6 ± 7.5	0.39	0.004	0.008
	L	41.5 ± 10.0	49.9 ± 6.8	0.44	0.001	0.002
Limbic	R	29.3 ± 4.6	30.6 ± 4.7	0.13	0.342	0.443
	L	28.4 ± 4.0	29.3 ± 4.3	0.07	0.611	0.707
Parietal	R	27.0 ± 7.4	39.0 ± 6.2	0.68	<0.001	<0.001
	L	25.1 ± 7.7	37.9 ± 6.3	0.69	<0.001	<0.001
Sub-lobar	R	29.7 ± 5.4	35.2 ± 5.3	0.47	<0.001	<0.001
	L	27.6 ± 4.4	32.2 ± 4.9	0.39	0.004	0.008
Occipital	R	18.5 ± 6.7	27.7 ± 3.9	0.64	<0.001	<0.001
	L	19.9 ± 6.6	29.3 ± 5.2	0.65	<0.001	<0.001
Midbrain	R	1.0 ± 0.2	1.2 ± 0.2	0.43	0.001	0.002
	L	0.7 ± 0.2	0.8 ± 0.2	0.27	0.045	0.083
Frontal–Temporal	R	0.1 ± 0.1	0.1 ± 0.1	0.12	0.371	0.453
	L	0.1 ± 0.1	0.1 ± 0.1	0.05	0.718	0.790
Cerebellum–Anterior	R	15.4 ± 2.5	16.6 ± 2.5	0.22	0.105	0.165
	L	14.6 ± 2.1	14.9 ± 2.1	0.02	0.870	0.911
Cerebellum–Posterior	R	17.8 ± 2.9	18.3 ± 2.3	0.01	0.954	0.954
	L	18.7 ± 3.4	20.1 ± 3.1	0.20	0.128	0.188
Pons	R	0.3 ± 0.1	0.4 ± 0.1	0.15	0.258	0.355
	L	0.3 ± 0.2	0.4 ± 0.1	0.25	0.064	0.108

CA: congenital anosmia. † Benjamini–Hochberg method.

**Table 4:** Comparison of white matter volumes between patients with CA and healthy controls.

Area	Patients with CA (cc)	Controls (cc)	Effect Size	*p*-Value	Adjusted *p*-Value †
Frontal	152 ± 19.9	139.2 ± 24	0.30	0.027	0.074
Temporal	71.5 ± 11.5	60.8 ± 9.5	0.45	0.001	0.004
Limbic	48.3 ± 7	48.3 ± 7.9	0.03	0.831	0.831
Parietal	75.8 ± 12.4	61.9 ± 11.5	0.50	<0.001	<0.001
Sub-lobar	79.6 ± 10.3	77.9 ± 13	0.10	0.441	0.485
Occipital	58.7 ± 10.3	41.9 ± 7.9	0.69	<0.001	<0.001
Midbrain	9.7 ± 1.6	10.6 ± 2	0.22	0.106	0.167
Frontal–Temporal	0.0 ± 0.0	0.0 ± 0.0	0.22	0.106	0.167
Cerebellum–Anterior	7.3 ± 1.9	7.7 ± 1.4	0.12	0.372	0.455
Cerebellum–Posterior	9.8 ± 2.7	8.5 ± 2.7	0.22	0.098	0.167
Pons	9 ± 1.2	9.3 ± 2	0.13	0.33	0.454

CA: congenital anosmia. † Benjamini–Hochberg method.

**Table 5:** Comparison of right/left white matter volumes between patients with CA and healthy controls.

Area	Hemisphere	Patients with CA (cc)	Controls (cc)	Effect Size	*p*-Value	Adjusted *p*-Value †
Frontal	R	75.8 ± 9.8	69.1 ± 12.4	0.30	0.024	0.072
	L	76.2 ± 10.4	70.0 ± 11.8	0.28	0.033	0.081
Temporal	R	32.9 ± 5.3	28.3 ± 4.5	0.41	0.002	0.007
	L	38.6 ± 6.4	32.5 ± 5.2	0.46	0.001	0.004
Limbic	R	27.8 ± 4.1	27.6 ± 4.6	0.03	0.819	0.858
	L	20.3 ± 3.2	20.3 ± 3.4	0.00	0.974	0.974
Parietal	R	36.0 ± 6.6	29.4 ± 5.3	0.48	<0.001	<0.001
	L	39.8 ± 6.1	32.6 ± 6.4	0.49	<0.001	<0.001
Sub-lobar	R	40.5 ± 5.4	39.1 ± 6.5	0.15	0.272	0.427
	L	38.4 ± 5.2	37.8 ± 6.4	0.09	0.502	0.575
Occipital	R	28.6 ± 4.8	20.1 ± 3.9	0.70	<0.001	<0.001
	L	29.9 ± 5.7	21.6 ± 4.1	0.65	<0.001	<0.001
Midbrain	R	4.9 ± 0.9	5.4 ± 1.1	0.19	0.154	0.308
	L	4.5 ± 0.8	4.9 ± 0.9	0.19	0.145	0.308
Frontal–Temporal	R	0.0 ± 0.0	0.0 ± 0.0	0.13	0.317	0.453
	L	0.0 ± 0.0	0.0 ± 0.0	0.17	0.199	0.365
Cerebellum–Anterior	R	3.8 ± 1.1	4.0 ± 0.7	0.09	0.523	0.575
	L	3.4 ± 0.9	3.6 ± 0.8	0.13	0.334	0.453
Cerebellum–Posterior	R	4.2 ± 1.4	3.8 ± 1.4	0.12	0.35	0.453
	L	5.6 ± 1.7	4.7 ± 1.6	0.30	0.026	0.072
Pons	R	4.4 ± 0.7	4.5 ± 1.0	0.16	0.245	0.415
	L	4.2 ± 0.5	4.3 ± 0.9	0.10	0.441	0.539

CA: congenital anosmia. † Benjamini–Hochberg method.

## Data Availability

The datasets used and analyzed in this study are available from the corresponding author upon reasonable request.

## Abbreviations

The following abbreviations are used in this manuscript:

BM	White matter
CA	Congenital anosmia
FLAIR	Fluid-attenuated inversion recovery
GM	Gray matter
MRI	Magnetic Resonance Imaging
PEA	Phenyl ethyl alcohol
